# Extraction of Polyphenols and Vitamins Using Biodegradable ATPS Based on Ethyl Lactate

**DOI:** 10.3390/molecules27227838

**Published:** 2022-11-14

**Authors:** Pedro Velho, Luís Marques, Eugénia A. Macedo

**Affiliations:** 1 LSRE-LCM—Laboratory of Separation and Reaction Engineering—Laboratory of Catalysis and Materials, Faculty of Engineering, University of Porto, Rua Dr. Roberto Frias, 4200-465 Porto, Portugal; 2 ALiCE—Associate Laboratory in Chemical Engineering, Faculty of Engineering, University of Porto, Rua Dr. Roberto Frias, 4200-465 Porto, Portugal

**Keywords:** ATPS, ethyl lactate, polyphenols, vitamins

## Abstract

The growing human population, together with the inefficient use of natural resources, has been dramatically increasing the production of food waste, which poses serious economic, environmental, and social problems. Being so, it is necessary to increase the efficiency of food consumption so as to reduce its waste and to convert the remaining residues into societal benefits. Since this biowaste is rich in polyphenols and vitamins, it could become the feedstock for the production of important value-added compounds for the pharmaceutical (*e.g.*, food supplements) and cosmetic (*e.g.*, creams and shampoos) industries. In this work, partition studies of one polyphenol (epicatechin) and two B-complex vitamins (cyanocobalamin and nicotinic acid) were performed in biodegradable Aqueous Two-Phase Systems (ATPS) based on ethyl lactate and on organic salts (disodium tartrate, tripotassium citrate, and trisodium citrate) at 298.15 K and 0.1 MPa. The largest partition coefficient (*K*) and extraction efficiency (E) were obtained for vitamin B12 (K=78.56, E=97.5%) for the longest tie line $\left(\mathrm{TLL}=77.66\%\right)$ in the ATPS {ethyl lactate (1) + tripotassium citrate (2) + water (3)}. All the extractions were obtained with low biomolecule mass losses in quantification (<5%) and after a thorough study of pH influence in the UV–Vis absorbance spectra.

## 1. Introduction

The steady growth of the human population, together with the inefficient use of natural resources, has been contributing to an increased production of food waste, which poses serious economic and social problems [1,2]. Furthermore, environmental issues such as farm-level water losses, land degradation, and greenhouse gas emissions from food distribution and decomposition are also linked to this global problem [2]. Being so, it is necessary to increase the efficiency of food consumption and to convert the remainder of this waste into societal benefits.

Successful attempts of using food waste as feedstock and contributing to a more circular economy are common in the literature, including its application in the production of fertiliser [3,4] and of insects for human food and animal feed [4]. Moreover, a particular type of sustainable biorefinery, the food waste biorefinery, has been gaining preponderance as a source of added-value compounds [5] and as a processual precursor of waste incineration and fertiliser production. This way, important biomolecules such as enzymes, peptides, fatty acids (*e.g.*, from fish processing wastes [5]), glucose, collagen, keratin (*e.g.*, from meat processing wastes [5]), vitamins, and polyphenols (*e.g.*, from fruit peals and pomaces [6,7]) are recovered and applied in the pharmaceutical and cosmetic industries and even reintroduced in the food sector [7,8].

Aqueous Two-Phase Systems (ATPS) or Aqueous Biphasic Systems (ABS) have emerged as a powerful tool for the recovery of biomolecules, for the analysis of cellular surfaces, and for the fractionation of cell populations [9]. Generally, ATPS are composed by a ternary mixture of water + polymers, water + polymer + salt, or water + organic solvent + salt. The presence of a salting-out agent (*e.g.*, salt or polymer) induces phase splitting and yields two liquid phases, known as top (lower density) and bottom (higher density) phases, with one of them being significantly richer in salting-out agent than the other. The distinct phase compositions create differences in properties such as hydrophobicity, polarity, and viscosity, which will rule solute migration (partition) between the phases. Since both their liquid phases are mostly composed by water, ATPS are gentle and biocompatible media for biomaterials and are characterised by low interfacial tensions when compared, for example, with conventional water/organic solvent systems [9,10].

Polyethylene glycol (PEG) is the most applied polymer for its nontoxicity, relative low-cost, and ease of synthesis [11], but product recovery is extremely hard and requires the handling of less eco-friendly solvents. PEG has been applied in the extraction of, for example, amino acids [12,13], active pharmaceutical ingredients (API) [14], flavonoids [15], and proteins [16].

Ethyl lactate is an environmentally benign bio-based green solvent with large solvating power. It is a hydrophilic, amphiphilic, bio-renewable, non-flammable, and biodegradable chemical with growing application as sustainable media for organic synthesis [17,18]. Further, it is also being used as a food additive, paint stripper, flavour chemical, and in perfumery [19,20]. Recently, ethyl lactate has been applied to extract biomolecules such as vitamins [21,22], pigments [22,23], antioxidants [24,25,26], amino acids [27], proteins [28], and antibiotics [29], replacing more hazardous solvents such as alcohols and dichloromethane.

Concerning salts, inorganic salts such as sulphates [30,31] and phosphates [30,32] have been extensively used in ATPS, but these may cause environmental distress at an industrial scale of operation [11]. Therefore, organic salts such as tartrates [12,30] and citrates [13,30] have been preferred due to their non-toxicity and biodegradability.

Recently, some novel ATPS have been developed to enforce the recyclability of their constituents and the selectivity for certain species, such as ATPS based on light-triggered switchable ionic liquids [33], on choline amino acid ionic liquids [34], and on surfactants and polyalcohols [35].

In this work, three biomolecules were studied, including one polyphenol (epicatechin) and two B-complex vitamins (cyanocobalamin and nicotinic acid).

Epicatechin (E) belongs to a particular category of polyphenols, the flavonoids, which are products of the secondary metabolism of plants [36]. Epicatechin is commonly found in a wide variety of foods, such as fruits (*e.g.*, apples, grapes, cherries, and apricots), vegetables, legumes, cocoa derivatives, wine, and some teas [37,38]. This catechin has been shown to reduce hypertension, to improve the endothelial function [39], and is thought to enhance cognition [40].

Cyanocobalamin or vitamin B12 is a corrinoid which can be found in red meat, milk, eggs, potatoes, and mushrooms [41]. It is essential for the normal functioning of the human body, especially to what concerns methylation and the mitochondrial metabolism, so its severe deficiency has been found to cause haematological (*e.g.*, megaloblastic anaemia) and neurological (*e.g.*, demyelination of the peripheral and central neurons) issues [42].

**Figure 1:**
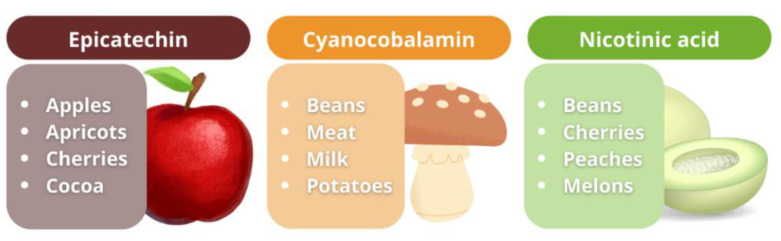
Possible feedstocks for the extraction of epicatechin [37,38], cyanocobalamin (vitamin B12) [41], and nicotinic acid [44].

Nicotinic acid (NA) is one of the forms of vitamin B3. It is a water-soluble vitamin and has been extensively used as a lipid-modifying drug due to its antidyslipidemic properties (counteracts the imbalance of lipids in blood) [43]. Significant quantities of nicotinic acid have been found in legumes and fruits, with chestnuts, figs, melons, peaches, and cherries being some of the most relevant [44]. Figure 1 summarizes the natural sources of the studied biomolecules in this work.

The aim of this work was to delve into the extraction of one polyphenol (epicatechin) and two vitamins (cyanocobalamin and nicotinic acid) so as to set the ground for future valorisation of food wastes (*e.g.*, vegetable peals and fruit pomaces) to value-added pharmaceutical (*e.g.*, food supplements) and cosmetic (*e.g.*, creams and shampoos) products. Different biodegradable and ethyl-lactate-based ATPS with organic salts were applied at 298.15 K and 0.1 MPa in the partition of these biomolecules for the first time, in an effort to find effective extraction media which could promote a more sustainable production of therapeutics and cosmetics and a more circular economy.

## 2. Materials and Methods

### 2.1. Chemicals

Table 1 presents the chemicals used in this work, together with their respective commercial suppliers, purities, Chemical Abstracts Service (CAS) number, and abbreviation. All the chemicals were used without any further purification step.

### 2.2. Apparatus and Experimental Procedure

In this work, an ADAM AAA 250L balance with measurement uncertainty of ±10^−4^ g was used to assess mass (m), and a Thermo Scientific Varioskan Flash spectrophotometer with measurement uncertainty of ±10^−4^ was used to determine UV–Vis absorbance (A). Further, temperature (T) was kept at 298.15 ± 0.01 K with a Julabo F12 thermostatic bath coupled with a Julabo ED controller, and density (ρ) was assessed using an Anton Paar DSA-5000M densimeter with measurement uncertainties of ±3 × 10^−5^ g·cm^−3^ in density and ±0.01 K in temperature. Lastly, pH was evaluated using a VWR pH1100L with measurement uncertainties of ±0.001 in pH and ±0.1 K in temperature.

#### 2.2.1. Influence of System’s pH in the UV–Vis Absorbance Spectra

To assess the influence of the phases’ pH in the UV–Vis absorbance spectra of the biomolecules, aqueous stock solutions with different pH values and with concentrations of about (1.54, 3.12, and 2.50) × 10^−4^ g·mL^−1^ were prepared for epicatechin, cyanocobalamin, and nicotinic acid, respectively. In the determinations, the maximum concentrations were defined by the solubility in water of the species and by the useful absorbance range of the spectrophotometer. The pH values of the solutions were adjusted by adding droplets of 0.5 M sodium hydroxide (NaOH) or 0.5 M acetic acid (CH_3_COOH) aqueous solutions. Afterwards, 200 μL samples of each solution were added to a Greiner bio-one polystyrene flat bottom plate, and an absorbance scanning was performed from 200 to 600 nm using the Thermo Scientific Varioskan Flash UV–Vis spectrophotometer, after having stabilised the samples at 298.15 K.

Moreover, to evaluate the stability of the UV–Vis absorbance spectra at different pH values, the solutions were left to settle for 3 days at 298.15 K without any especial protection from daylight, after which a new absorbance screening was conducted with the UV–Vis spectrophotometer following the same procedure. These two spectra were then compared to evaluate the stability of the UV–Vis spectra at the different pH values.

#### 2.2.2. UV–Vis Absorbance Calibration Curves

To determine adequate UV–Vis absorbance calibration curves, 2 mL aqueous mixtures with different concentrations of biomolecule (epicatechin, cyanocobalamin, or nicotinic acid) were prepared in vials by diluting fresh stock solutions at a pH value of ~7.5, which was considered close to the characteristic pH of all the different ATPS used. After being capped and sealed with parafilm, the vials were vigorously stirred in a VWR VV3 vortex for about 2 min and in an IKA RO 10 P magnetic stirrer for 20 min. Afterwards, 200 μL samples of each vial were taken to the Thermo Scientific Varioskan Flash UV–Vis spectrophotometer, and an absorbance scanning, from 200 to 600 nm, was performed following the previously explained procedure. Then, the UV–Vis calibration curves were determined by plotting the biomolecules’ concentrations with the absorbances at a chosen wavelength and fitting the data to a first-degree equation after having subtracted the absorbance of blanks. The chosen wavelengths were 278, 363, and 264 nm for epicatechin, cyanocobalamin, and nicotinic acid, respectively. The absorbance of eventual pH adjusters (NaOH or CH_3_COOH) was considered negligible.

#### 2.2.3. Liquid–Liquid Extraction of Biomolecules

Vials with mixtures of 10 mL were prepared corresponding to the known tie lines (isothermal lines which connect two corresponding phases) of the ATPS {ethyl lactate (1) + disodium tartrate (2) + water (3)} [24], {ethyl lactate (1) + trisodium citrate (2) + water (3)} [25], and {ethyl lactate (1) + tripotassium citrate (2) + water (3)} [25], which were determined in previous works of the research group. These mixtures were prepared by pipetting and weighing the pure compounds (water and ethyl lactate) and the aqueous solutions of the organic salts: disodium tartrate (30.00 *m*%), trisodium citrate (25.59 *m*%), and tripotassium citrate (32.75 *m*%). In the preparation of the mixtures, 1 mL of the reported water content in [24,25] was replaced by 1 mL of stock solution of the biomolecule being studied (epicatechin, cyanocobalamin, or nicotinic acid). After being stirred in a vortex for 2 min, the samples were left under stirring for 6 h in the Julabo F12 thermostatic bath at 298.15 K. Then, the vials were left settling overnight (~12 h) at the same temperature. Afterwards, the top and bottom phases were carefully removed using pipettes and weighed in the ADAM AAA 250L balance. Moreover, the UV–Vis absorbances, the pH values, and the densities of the two phases of each tie line were assessed, by this order, with the Thermo Scientific Varioskan Flash spectrophotometer, VWR pH 1100 L pH meter, and Anton Paar DSA-5000M densimeter, respectively. The densimeter was cleaned between measurements with water and ethanol.

## 3. Results and Discussion

Due to the lability of vitamins and antioxidant species (such as polyphenols), their chemical formula/conformation can be changed by the pH of the liquid phase, causing the appearance of new chemical compounds. The differently charged species that a biomolecule may present, also known as stages [21], generally show different affinities to the ATPS phases, so properly identifying the stages which are present in a solution is essential to study biomolecule-oriented extractive processes.

Since the decimal logarithms of the dissociation constants $\left(pK_{a}\right)$ of epicatechin (8.72, 9.49, 11.23, and 13.40 [45]), cyanocobalamin (3.28 [46]), and nicotinic acid (2.00 and 4.82 [47]) are available in literature, the ratios between two successive biomolecule stages, *i.e.*, between a biomolecule of a certain charge and its closest reduced state, can be determined as a function of pH using Equation (1) [21]:

(1) $$\frac{\left[S^{q_{0}-\left(i-1\right)}\right]}{\left[S^{q_{0}-i}\right]}=10^{{\mathrm{pH}}_{\mathrm{phase}}-pK_{a}^{i}}$$

where $q_{0}$ is the charge of the antioxidant at pH=0, i is the number of the dissociation constant ($pK_{a}^{i}$) being considered, ${\mathrm{pH}}_{\mathrm{phase}}$ is the pH of the phase under study, and $\left[S^{q_{0}-\left(i-1\right)}\right]$ and $\left[S^{q_{0}-i}\right]$ are the molar concentrations of the biomolecule stages with electrical charges of $q_{0}-\left(i-1\right)$ e and $q_{0}-i$ e, respectively. e stands for the elementary charge (1.602 ∙ 10^−19^ C).

Then, the mean electrical charge of the antioxidant $\left(q\right)$ can be calculated using a weighted arithmetic mean:

(2) $$q=\overset{i_{\max}}{\underset{i=1}{\sum }}\left[x_{S^{q_{0}-\left(i-1\right)}}\times \left(q_{0}-\left(i-1\right)\right)\right]$$

where $x_{S^{q_{0}-\left(i-1\right)}}$ is the fraction (relative abundance) of the biomolecule stage with an electrical charge equal to $q_{0}-\left(i-1\right)$ e.

**Figure 2:**
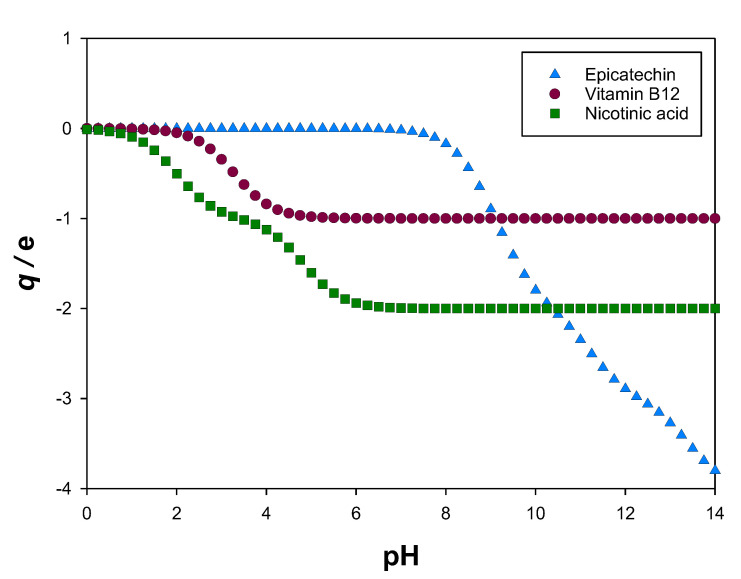
Calculated mean electrical charge $\left(q\right)$ for epicatechin, cyanocobalamin (vitamin B12), and nicotinic acid, expressed in terms of the elementary charge (e), *i.e.*, 1.602 × 10^−19^ C.

In Figure 2, the calculated mean electrical charges $\left(q\right)$ at different pH values for the studied biomolecules (epicatechin, cyanocobalamin, and nicotinic acid) can be seen.

In this work, the partition studies of epicatechin, cyanocobalamin, and nicotinic acid were performed in the ATPS shown in Table 2. According to the available literature [24,25], the phase separation of these ATPS causes pH values from 6 to 8. As observed in Figure 2, in this range, the mean electrical charges $\left(q\right)$ of these biomolecules correspond almost entirely to an integer value, so only one species (stage) is present, which allows better characterisation of the final extract. In Tables S1–S3 in the Supplementary Materials, the fractions of each biomolecule stage ($x_{S}$) for the data shown in Figure 2 can be observed. There, it can be noticed that, for example, at pH = 7.5, the molar fractions of the most common stages of epicatechin (E), cyanocobalamin (B12), and nicotinic acid (NA) are, respectively, $x_{E^{0}}=0.94$, $x_{B12^{-1}}=1.00$, and $x_{{\mathrm{NA}}^{-2}}=1.00$.

### 3.1. Influence of pH in the UV–Vis Absorbance Spectra

**Figure 3:**
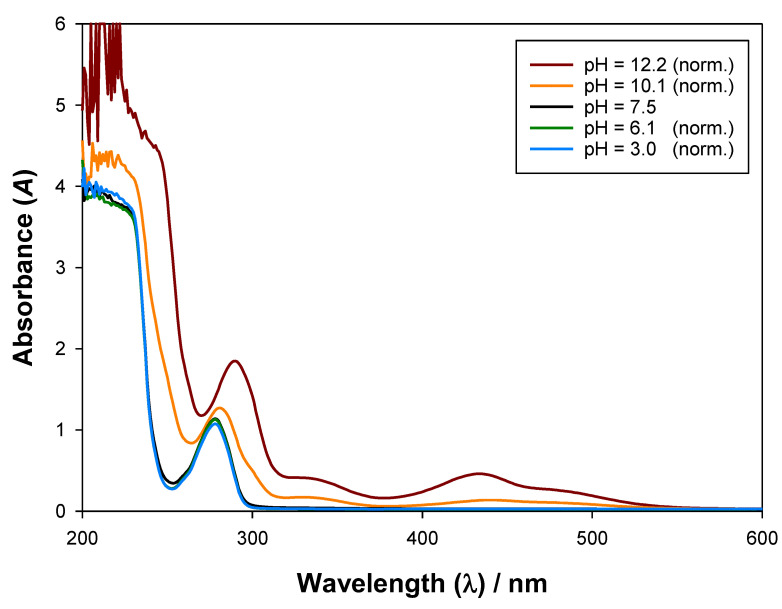
Influence of pH in the UV–Vis absorbance spectra of epicatechin (3.12 × 10^−4^ g·mL^−1^) at 298.15 K and 0.1 MPa.

As previously stated, the relative abundance of each biomolecule stage is heavily influenced by pH, since its variation leads to changes in the chemical structure and/or chemical conformation. Being so, the UV–Vis absorbance spectrum may also be altered, for which studying the influence of pH will be crucial for accurately determining the calibration curves. Therefore, absorbance measurements were performed from 200 to 600 nm in aqueous solutions with different pH values and with concentrations of about (1.54, 3.12, and 2.50) × 10^−4^ g·mL^−1^ for epicatechin (Figure 3), cyanocobalamin (Figure S1), and nicotinic acid (Figure S2), respectively. These absorbance spectra were normalised, having in consideration the amount of pH adjusters (0.5 M NaOH and 0.5 M CH_3_COOH) added using Equation (3), so as to ease interpretation.

(3) $$A^{\prime }=A\cdot \frac{C_{\mathrm{pH}=7.5}}{C_{\mathrm{pH}=k}}$$

Here, A is the experimental UV–Vis absorbance for a given wavelength $\left(\lambda \right)$, A′ is the normalised absorbance, $C_{\mathrm{pH}=7.5}$ is the reference concentration $\left(\mathrm{pH}=7.5\right)$, and $C_{\mathrm{pH}=k}$ is the concentration of the stock solution of biomolecule at a given pH k.

As Figure 3 shows for epicatechin, larger pH values imply larger UV–Vis absorbances and a progressively higher wavelength for the local maximum verified at 278 nm (and pH = 7.5). For cyanocobalamin (Figure S1) and nicotinic acid (Figure S2), the found spectra were almost independent of pH, hinting remarkably similar absorbance for all their biomolecule stages, which generally ensures more precise quantification of their concentrations using UV–Vis absorbance calibration curves.

### 3.2. UV–Vis Absorbance Calibration Curves

**Figure 4:**
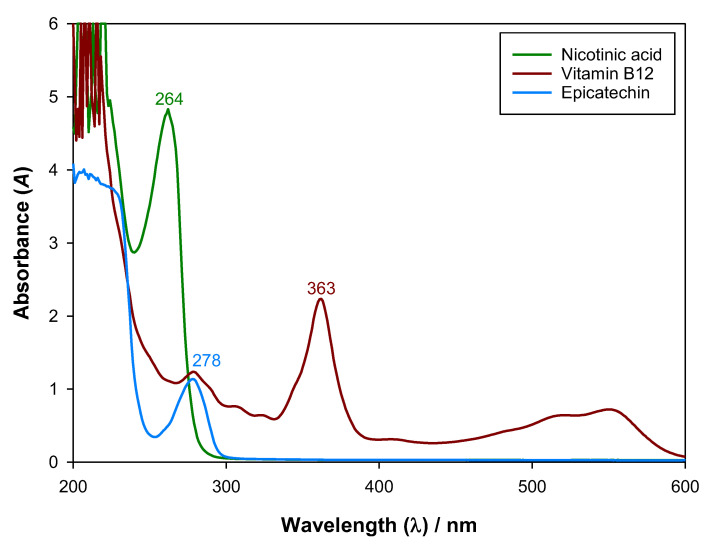
UV–Vis absorbance spectra at pH = 7.5, from 200 to 600 nm, for epicatechin, cyanocobalamin (vitamin B12), and nicotinic acid at (1.54, 3.12, and 2.50) × 10^−4^ g·mL^−1^, respectively, with T = 298.15 K and P = 0.1 MPa.

**Figure 5:**
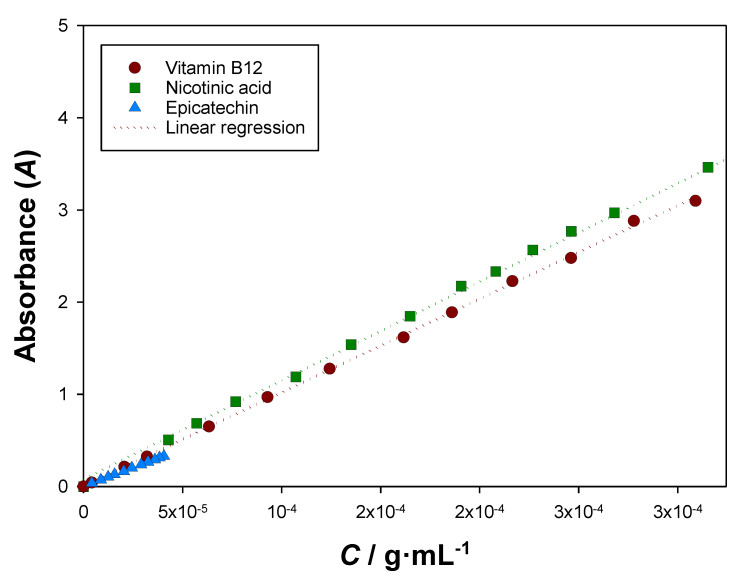
UV-Vis absorbance calibration curves at pH = 7.5 for epicatechin (278 nm), vitamin B12 (363 nm), and nicotinic acid (264 nm), with T = 298.15 K and P = 0.1 MPa. The first-degree fittings follow equations: $A=8091.2\times C_{E}\left(g\cdot {\mathrm{mL}}^{-1}\right)+0.0027$ with a determination coefficient ($R^{2}$) of 0.9998, $A=10024\times C_{B12}\left(g\cdot {\mathrm{mL}}^{-1}\right)+0.0123$ with $R^{2}=0.9998$, and $A=10630\times C_{\mathrm{NA}}\left(g\cdot {\mathrm{mL}}^{-1}\right)+0.1000$ with $R^{2}=0.9993$, respectively.

To enable an adequate quantification of the biomolecules after phase separation, UV–Vis calibration curves were determined at the wavelengths of 278, 363, and 264 nm for epicatechin, cyanocobalamin, and nicotinic acid, respectively. The UV–Vis absorbance spectra of the studied biomolecules can be seen in Figure 4. The calibration curves were conducted at a pH value close to the ones available in the literature for the studied ATPS $\left(\mathrm{pH}\approx 7.5\right)$ and can be observed in Figure 5. In the determinations, the absorbance of water (and plate) was subtracted, and the calibration curves were determined at the wavelengths that corresponded to the local or global maxima in which the other ATPS species (ethyl lactate and organic salts) and pH adjusters (NaOH and CH_3_COOH) did not significantly interfere.

Furthermore, since partition determinations take around 18 h (6 h of stirring and 12 h of settling), it is essential for the UV–Vis absorbance spectra to remain constant so as to be able to apply the found calibration curves (Figure 5). Being so, the prepared aqueous solutions of the studied biomolecules were left settling for three days without any especial protection from daylight, and their absorbance spectra were measured and compared to the initial ones. As Figures S3–S5 in the Supplementary Materials show, the chosen absorbance maxima were not affected by 3 days of settling, so their usage after ~18 h was validated.

### 3.3. Partitioning of Biomolecules

The applied ATPS were studied in previous works of the research group [24,25], so their liquid–liquid equilibria (LLE) were not determined in this work, and the partitioning of biomolecules was conducted at 298.15 K and 0.1 MPa in the previously reported tie-lines, which can be observed in Table 3.

So as to quantify the partition of the studied biomolecules, after phase equilibrium was reached, the liquid phases were separated and mass (*m*), absorbance (*A*), pH, and density (*ρ*) were measured. Then, the mass losses ($L_{m}$) were calculated, in percentage, using Equation (4). The results are shown in Table 4.

(4) $$L_{m}/\%=\frac{m_{2}-m_{1}}{m_{1}}\times 100$$

Here, $m_{1}$ is the total mass (feed), and $m_{2}$ is the sum of masses of the two phases separated after equilibrium was reached.

As seen in Table 4, UV-Vis absorbances were always higher for top phases, so all the studied biomolecules preferentially diffused into the ethyl-lactate-rich phase. Generally, the measured pH and density values of the phases were larger for the ATPS containing tripotassium citrate and smaller for the ATPS based on disodium tartrate. Moreover, pH values for the phases of each tie line in each system were alike, which implies similar distribution of electrical charges and, consequently, similar mean electrical charge $\left(q\right)$ in both phases of the same tie-line composition. This way, the phases presented homogeneous characteristics and a single calibration curve could be applied.

**Figure 6:**
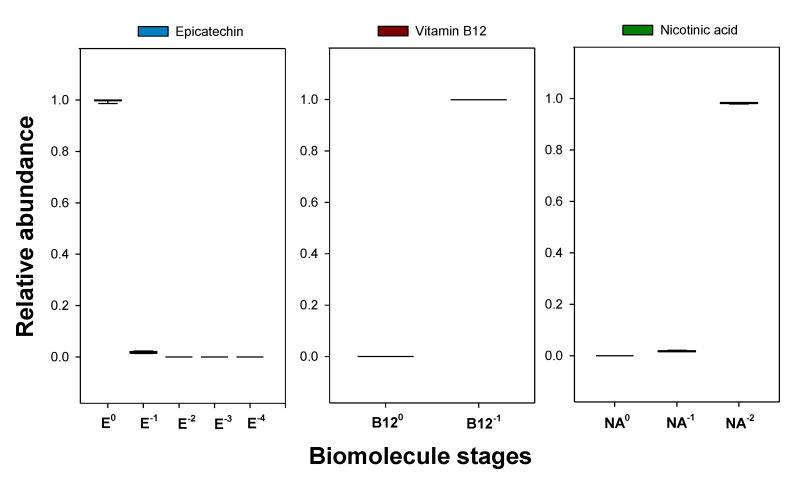
Influence of the tie-line compositions in the fraction of the biomolecule stages of epicatechin, cyanocobalamin, and nicotinic acid in the ATPS {ethyl lactate (1) + disodium tartrate (2) + water (3)} at 298.15 K and 0.1 MPa. E^0^, E^−1^, E^−2^, E^−3^, and E^−4^ stand for the biomolecule stages of epicatechin with electrical charges equal to 0, −1, −2, −3, and −4 e, respectively; B12^0^ and B12^−1^ stand for the biomolecule stages of cyanocobalamin with electrical charges equal to 0 and −1 e, respectively; NA^0^, NA^−1^, and NA^−2^ stand for the biomolecule stages of nicotinic acid with electrical charges equal to 0, −1, and −2 e, respectively, and e stands for the elementary charge (1.602×10^−19^ C).

Since using different tie-line compositions caused different pH values in the liquid phases (as seen in Table 4), it was necessary to understand if the distribution of biomolecule stages was similar between phases of different tie-line composition (for the same system). As Figure 6 shows, the ATPS {ethyl lactate (1) + disodium tartrate (2) + water (3)} ensured similar and well-defined stages for all the biomolecules, so its tie-line compositions yielded a homogenous biomolecule extract. The same conclusion can be drawn from {ethyl lactate (1) + trisodium citrate (2) + water (3)} in extracting nicotinic acid and from {ethyl lactate (1) + tripotassium citrate (2) + water (3)} in extracting cyanocobalamin, as Figures S6 and S7 in the Supplementary Materials, respectively, show. However, as can be seen in Figure S7 in the Supplementary Materials, the different tie-line compositions of {ethyl lactate (1) + tripotassium citrate (2) + water (3)} caused different distributions of biomolecule stages between the tie lines for epicatechin, particularly to what concerns the neutral and mono negatively charged species, originating more heterogeneous extracts.

With the measured UV-Vis absorbances and with the determined calibration curves (Figure 5), after subtracting the blanks, the concentrations of each biomolecule in each ATPS were determined, and a partition coefficient (K) was determined for each tie-line composition using Equation (5). These results can be observed in Table 5.

(5) $$K_{i}=\frac{C_{i}^{\mathrm{top}}}{C_{i}^{\mathrm{bottom}}}$$

Here, i is the tie-line number, and $C_{i}^{\mathrm{top}}$ and $C_{i}^{\mathrm{bottom}}$ correspond to the biomolecule’s concentration in the top and bottom phases, respectively.

As can be observed in Table 5, the extraction of vitamin B12 in the ATPS {ethyl lactate (1) + K_3_Citrate (2) + water (3)} presented the largest partition coefficients, reaching K = 78.56 with TLL = 77.66%. Conversely, nicotinic acid in {ethyl lactate (1) + Na_2_Tartrate (2) + water (3)} yielded the worst results, obtaining K = 2.60 for the longest tie line (TLL = 70.90%). Moreover, all the systems achieved partition coefficients above unity, which means that the top phases’ concentrations of biomolecule were always larger than the ones of the bottom phases. Therefore, ethyl lactate, which is mostly present in the top phases, was successful in extracting the biomolecules.

**Figure 7:**
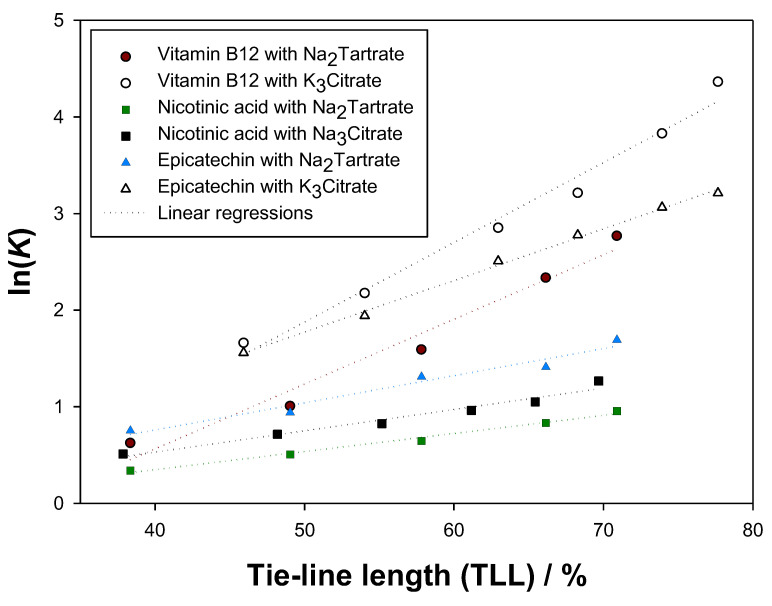
Relation of the tie-line length (TLL) [24,25] with the natural logarithm of the experimental partition coefficients (*K*) in the ATPS {ethyl lactate (1) + Na_2_Tartrate or Na_3_Citrate or K_3_Citrate (2) + water (3)} at 298.15 K and 0.1 MPa for epicatechin, vitamin B12, and nicotinic acid.

Furthermore, as Figure 7 shows, all biomolecules suffered an increase in the top phase concentration for longer tie lines, *i.e.*, for more distinct top and bottom compositions, so ethyl lactate presents good affinity for these species. The more positive the slope of the lines, the more favoured solute migration for the top phase is with growing tie-line length, so ethyl lactate presents more affinity for cyanocobalamin. Generally, a close to linear behaviour was verified for the natural logarithm of the partition coefficients (ln$\left(K\right)$) with the tie-line lengths (TLL).

### 3.4. Mass Balance

To ensure the validity of the reported partition coefficients of Table 5, it is of the utmost importance to validate the analytical method by performing a mass balance on the biomolecules under study, *i.e.*, verifying that all the biomolecule mass is being considered. First, the liquid volumes (V) of all the phases (top and bottom) were determined using:

(6) $$V_{j}=\frac{m_{j}}{\rho_{j}}$$

where $V_{j}$ is the liquid phase volume, $m_{j}$ is the measured mass, and $\rho_{j}$ is the measured density for phase *j.*

Then, the mass balance was checked by calculating the solute losses ($L_{s}$) using:

(7) $$L_{s}/\%\;=\frac{m_{s2}-m_{s1}}{m_{s1}}\times 100$$

where $m_{s1}$ is the added mass of biomolecule (present in 1 mL of stock solution), and $m_{s2}$ is the total quantified mass of biomolecule, which was calculated using:

(8) $$m_{s2}=V_{i}^{\mathrm{top}}C_{i}^{\mathrm{top}}+V_{i}^{\mathrm{bottom}}C_{i}^{\mathrm{bottom}}$$

where $V_{i}^{\mathrm{top}}$ and $V_{i}^{\mathrm{bottom}}$ are the calculated experimental volumes of the top and bottom phases, respectively, and i refers to the tie-line number.

Further, the extraction efficiencies of each tie line (E) were determined with:

(9) $$E/\%=\frac{m_{\mathrm{top}}}{m_{s1}}\times 100$$

where $m_{\mathrm{top}}$ is the quantified mass of biomolecule in the top phase.

Since the solute losses quantified using Equation (7) may be in the top phase, an extraction efficiency interval can be found using the values determined for E as the minimum boundary and summing the absolute value of $L_{s}$ with E for the maximum limit. All these results are presented in Table 6.

**Figure 8:**
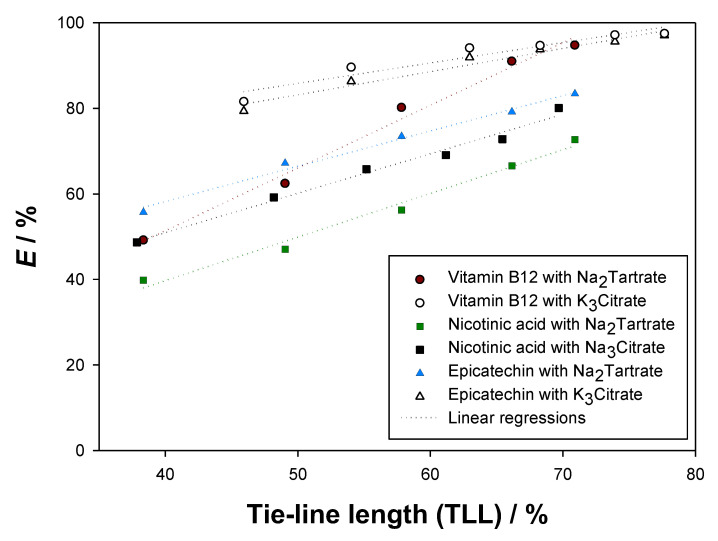
Relation of the tie-line length (TLL) [24,25] with the extraction efficiencies $\left(E\right)$ in the ATPS {ethyl lactate (1) + Na_2_Tartrate or Na_3_Citrate or K_3_Citrate (2) + water (3)} at 298.15 K and 0.1 MPa for epicatechin, cyanocobalamin, and nicotinic acid.

As Table 6 shows, low solute losses were obtained for epicatechin (<5%), vitamin B12 (<3%), and nicotinic acid (<5%). Therefore, the validity of the analytical method was assured, confirming the reported partition coefficients (K) and extraction efficiencies (E). Moreover, vitamin B12 presented the largest extraction efficiencies, and E increased with growing tie-line length (TLL) for all the studied biomolecules. In Figure 8, the extraction efficiencies $\left(E\right)$ were plotted in the function of the tie-line lengths (TLL) to ease comparison.

As seen in Figure 8, the longest tie-lines provided the highest extraction efficiencies (E). Therefore, solute migration to the top phases was favoured by more distinct compositions of the phases, *i.e.*, higher concentration of ethyl lactate in the top phases and higher salt concentration in the bottom phases. Epicatechin and vitamin B12 reached extraction efficiencies close to 100%, while nicotinic acid achieved, at maximum, 81.7%.

By observing Figure 7 and Figure 8, it can be concluded that the best system for the extraction of vitamin B12 (cyanocobalamin) at 298.15 K and 0.1 MPa is {ethyl lactate (1) + K_3_Citrate (2) + water (3)}, since it yields larger partition coefficients (presents a more top phase-centred solute distribution) and larger extraction efficiencies (top phases retain a more significant fraction of the added biomolecule) than {ethyl lactate (1) + Na_2_Tartrate (2) + water (3)}. Following the same logic, {ethyl lactate (1) + K_3_Citrate (2) + water (3)} is better than {ethyl lactate (1) + Na_2_Tartrate (2) + water (3)} at extracting epicatechin, and {ethyl lactate (1) + Na_3_Citrate (2) + water (3)} provides more reliable extractive media for nicotinic acid than {ethyl lactate (1) + Na_2_Tartrate (2) + water (3)}. These findings follow the generally observed trend that citrate-based organic salts ensure more efficient extractions of biomolecules than tartrate-based organic salts.

## 4. Conclusions

Reducing food waste and converting it to societal benefits has become a topic of great importance due to the exponential growth of the human population and the inefficient use of natural resources. Biomolecules such as polyphenols (*e.g.*, epicatechin) and vitamins (*e.g.*, cyanocobalamin and nicotinic acid) are present in some vegetables, fruits, and legumes, for which their presence in food waste is inevitable. These chemical species conceal unique nutritive and medicinal properties, so they have been added to pharmaceuticals (*e.g.*, food supplements) and cosmetics (*e.g.*, creams and shampoos).

In this work, partition studies of epicatechin, vitamin B12 (cyanocobalamin), and nicotinic acid were successfully conducted in the ATPS {ethyl lactate (1) + Na_2_Tartrate or Na_3_Citrate or K_3_Citrate (2) + water (3)} at 298.15 K and 0.1 MPa for future valorisation of food wastes such as vegetable peals and fruit pomaces. The largest partition coefficients (*K*) and extraction efficiencies (E) were obtained for vitamin B12 (K=78.56, E=97.5%) and for epicatechin (K=24.86, E=97.0%) for the longest tie line $\left(\mathrm{TLL}=77.66\%\right)$ in the ATPS {ethyl lactate (1) + tripotassium citrate (2) + water (3)}. Therefore, this is the most efficient extractive system for future valorisation of vitamin-B12-rich (*e.g.*, potato peals) and epicatechin-rich (*e.g.*, apple peals) food waste.

All the applied ATPS provided partition coefficients larger than unity, for which they were considered successful in the extraction of the studied biomolecules. The reported extraction efficiencies (E) and partition coefficients (K) were validated by the verified low biomolecule mass losses in quantification for vitamin B12 (<3%), epicatechin (<5%), and nicotinic acid (<5%), which were achieved after a thorough study of the influence of pH in the UV–Vis absorbance spectra of these biomolecules.

## Figures and Tables

**Table 1:** Chemicals used in this work, with respective chemical formula, suppliers, purities, CAS number, and abbreviation.

Chemical	Supplier	Purity ^a^/*m*% ^b^	CAS	Abbreviation
Acetic acid (CH_3_COOH)	Merck	>99.8	64-19-7	-
Cyanocobalamin or vitamin B12 (C_63_H_88_CoN_14_O_14_P)	Sigma-Aldrich	>98	68-19-9	B12
(-)-epicatechin (C_15_H_14_O_6_)	Tokyo Chemical Industry	>97	490-46-0	E
Ethanol (CH_3_CH_2_OH)	Sigma-Aldrich	>99	64-17-5	-
(-)-ethyl L-lactate (C_5_H_10_O_3_)	Sigma-Aldrich	>98	97-64-3	EL
Nicotinic acid (C_6_H_5_NO_2_)	Sigma-Aldrich	>99.5	59-67-6	NA
Purified water (H_2_O)	VWR chemicals	-	7732-18-5	W
Sodium hydroxide (NaOH)	Merck	>99	1310-73-2	-
Potassium citrate monohydrate (C_6_H_5_K_3_O_7_·H_2_O)	Sigma-Aldrich	>99	6100-05-6	K_3_Citrate
Sodium citrate tribasic dihydrate (C_6_H_5_Na_3_O_7_·2H_2_O)	Sigma-Aldrich	>99	6132-04-3	Na_3_Citrate
Sodium tartrate dihydrate (C_4_H_4_Na_2_O_6_·2H_2_O)	VWR chemicals	>99.9	6106-24-7	Na_2_Tartrate

^a^ Provided by the supplier; ^b^ *m*% refers to mass percentage.

**Table 2:** Extracted biomolecules using the Aqueous Two-Phase Systems (ATPS) {ethyl lactate (1) + organic salt (2) + water (3)}.

Biomolecule	Organic Salts		
	Na_2_Tartrate	Na_3_Citrate	K_3_Citrate
Epicatechin	×		×
Cyanocobalamin	×		×
Nicotinic acid	×	×	

**Table 3:** Determined tie-lines for the ATPS {ethyl lactate (1) + organic salt (2) + water (3)} used in this work at 298.15 K and 0.1 MPa ^a,b^ [24,25].

Tie Line	Feed		Phase	Separation		
	$w_{1}$/*m*%	$w_{2}$/*m*%		$w_{1}$/*m*%	$w_{2}$/*m*%	pH
	{ethyl lactate (1) + disodium tartrate (2) + water (3)} [25]					
**1**	28.0	12.5	Top	51.4	3.7	6.18
			Bottom	15.5	17.0	6.10
**2**	30.0	13.0	Top	57.5	2.7	6.13
			Bottom	11.6	19.8	6.17
**3**	32.5	13.3	Top	63.1	2.0	6.13
			Bottom	9.0	22.3	6.18
**4**	35.5	13.8	Top	68.9	1.5	6.15
			Bottom	7.0	24.8	6.18
**5**	38.0	14.0	Top	72.4	1.3	6.11
			Bottom	6.0	26.2	6.17
	{ethyl lactate (1) + trisodium citrate (2) + water (3)} [24]					
**1**	30.0	11.0	Top	51.7	3.0	7.00
			Bottom	16.0	15.7	6.98
**2**	32.0	11.4	Top	57.5	2.0	6.98
			Bottom	12.3	18.5	6.96
**3**	34.3	11.7	Top	61.5	1.4	6.98
			Bottom	9.8	20.7	6.97
**4**	36.5	12.1	Top	65.0	1.0	7.00
			Bottom	7.9	23.0	7.00
**5**	38.5	12.3	Top	67.7	0.7	6.98
			Bottom	6.8	24.7	6.97
**6**	40.6	12.6	Top	70.1	0.5	6.98
			Bottom	5.5	26.6	7.00
	{ethyl lactate (1) + tripotassium citrate (2) + water (3)} [24]					
**1**	35.5	12.6	Top	57.9	3.9	7.21
			Bottom	15.0	20.3	7.39
**2**	37.5	13.0	Top	61.7	3.4	7.22
			Bottom	11.5	23.2	7.41
**3**	39.2	13.5	Top	67.4	2.1	7.19
			Bottom	9.1	25.8	7.37
**4**	41.1	13.9	Top	70.4	1.6	7.23
			Bottom	7.5	28.2	7.41
**5**	43.0	14.3	Top	73.6	1.1	7.12
			Bottom	6.0	31.0	7.39
**6**	44.6	14.8	Top	75.8	0.9	7.22
			Bottom	5.2	33.1	7.43

^a^ $w_{i}$ stands for the mass percentage (*m*%) of species i.; ^b^ standard uncertainties $\left(u\right)$ are: $u\left(T\right)$ = 0.2 K, $u\left(P\right)$ = 10 kPa, $u\left(w_{i}\right)$ = 10^−1^, and $u\left(\mathrm{pH}\right)$ = 10^−2^ [24,25].

**Table 4:** Experimental mass (m), UV-Vis absorbance (A) at chosen wavelength $\left(\lambda \right)$, density (ρ), pH, and mass loss ($L_{m}$) for the top and bottom phases in the extraction of epicatechin, cyanocobalamin, and nicotinic acid using the ATPS {ethyl lactate (1) + Na_2_Tartrate or Na_3_Citrate or K_3_Citrate (2) + water (3)} at 298.15 K and 0.1 MPa ^a^.

Tie Line	Phase	m/g	$L_{m}$/%	*A*	*ρ*/g·mL^−1^	pH
		**Epicatechin (λ=278 nm)–Na_2_Tartrate**				
**1**	Top	3.6786	−0.48	0.8346	1.05790	6.880
	Bottom	6.4186		0.4077	1.12550	6.862
**2**	Top	4.4509	−0.10	0.8870	1.04840	6.996
	Bottom	5.5871		0.3671	1.13730	6.873
**3**	Top	4.3920	−1.95	0.9332	1.04750	7.014
	Bottom	5.4675		0.3379	1.15600	6.896
**4**	Top	4.6811	−0.74	0.9686	1.04550	7.049
	Bottom	5.3176		0.3339	1.17670	7.078
**5**	Top	5.1993	−1.92	0.9896	1.04210	7.083
	Bottom	4.6718		0.3297	1.19110	6.986
		**Epicatechin (λ=278 nm)–K_3_Citrate**				
**1**	Top	4.4828	−0.19	0.8568	1.05867	8.215
	Bottom	5.5222		0.2778	1.14870	8.248
**2**	Top	4.8564	−1.41	0.8888	1.05425	8.285
	Bottom	5.0553		0.2320	1.16630	8.283
**3**	Top	5.1306	−1.44	0.9502	1.05210	8.244
	Bottom	4.8115		0.2056	1.17672	8.321
**4**	Top	5.2578	−0.85	0.9688	1.04671	8.266
	Bottom	4.8111		0.1992	1.19459	8.485
**5**	Top	5.4584	−1.17	0.9984	1.04583	8.291
	Bottom	4.5713		0.1882	1.21058	8.509
**6**	Top	5.7649	−1.65	1.0392	1.04572	8.344
	Bottom	4.3962		0.1811	1.21871	8.515
		**Vitamin B12 (λ=363 nm)–Na_2_Tartrate**				
**1**	Top	3.3444	−1.39	2.0127	1.05920	6.990
	Bottom	6.6009		1.1287	1.12560	6.905
**2**	Top	3.8218	−1.39	2.2431	1.06760	7.010
	Bottom	6.0977		0.8812	1.10820	6.958
**3**	Top	4.5850	−1.36	2.3748	1.05170	6.952
	Bottom	5.3460		0.5605	1.12960	7.063
**4**	Top	5.1427	−0.49	2.3930	1.04900	6.965
	Bottom	4.8684		0.3193	1.14630	7.063
**5**	Top	5.4989	−0.48	2.3171	1.04580	6.988
	Bottom	4.5034		0.2393	1.16320	7.150
		**Vitamin B12 (λ=363 nm)–K_3_Citrate**				
**1**	Top	4.6756	−1.29	2.3753	1.05970	8.103
	Bottom	5.2595		0.5091	1.15070	8.068
**2**	Top	5.0931	−0.91	2.3588	1.05330	8.141
	Bottom	4.8615		0.3312	1.16280	8.097
**3**	Top	5.1493	−0.88	2.4601	1.05020	8.188
	Bottom	4.8149		0.2024	1.17200	8.108
**4**	Top	5.2956	−1.17	2.4048	1.04800	8.206
	Bottom	4.6424		0.1611	1.19390	8.156
**5**	Top	5.418	−0.97	2.4107	1.04550	8.233
	Bottom	4.5434		0.1183	1.20940	8.191
**6**	Top	5.5163	−1.15	2.3630	1.04470	8.281
	Bottom	4.4272		0.0965	1.21970	8.263
		**Nicotinic acid (λ=264 nm)–Na_2_Tartrate**				
**1**	Top	3.0821	−1.03	4.0890	1.05322	6.619
	Bottom	6.8828		2.7974	1.12553	6.531
**2**	Top	3.4818	−0.64	4.3159	1.05680	6.629
	Bottom	6.6076		2.5573	1.12730	6.529
**3**	Top	3.9679	−1.31	4.5031	1.05280	6.638
	Bottom	5.9567		2.3235	1.13440	6.496
**4**	Top	4.5769	−1.24	4.6137	1.04670	6.568
	Bottom	5.3389		2.0370	1.15830	6.539
**5**	Top	5.0313	−1.09	4.6525	1.04640	6.644
	Bottom	4.9116		1.8679	1.17280	6.618
		**Nicotinic acid (λ=264 nm)–Na_3_Citrate**				
**1**	Top	3.6279	−0.76	4.0313	1.05381	7.644
	Bottom	6.4795		2.3153	1.12229	7.534
**2**	Top	4.0828	−0.21	4.3514	1.04788	7.748
	Bottom	5.8773		2.0339	1.13681	7.554
**3**	Top	4.5328	−0.21	4.3578	1.04558	7.685
	Bottom	5.4912		1.8313	1.15010	7.551
**4**	Top	4.7552	−0.48	4.3915	1.04354	7.735
	Bottom	5.2350		1.6074	1.16720	7.603
**5**	Top	4.9717	−0.76	4.4329	1.04209	7.721
	Bottom	4.9846		1.4982	1.18528	7.621
**6**	Top	5.1887	−0.42	4.1538	1.04118	7.851
	Bottom	4.8186		1.3575	1.19160	8.048

^a^ The measurement uncertainties $\left(u\right)$ are: $u\left(m\right)=10^{-4}\;g$, $u\left(A\right)=10^{-4}$, $u\left(\rho \right)=3\times 10^{-5}\;g\cdot \mathrm{mL}\;$ and $u\left(\mathrm{pH}\right)=10^{-3}$.

**Table 5:** Calculated concentration of biomolecule (*C*) for each phase, partition coefficients (*K*) for each tie-line composition, and tie-line lengths (TLL) for the extraction of epicatechin, cyanocobalamin, and nicotinic acid using the ATPS {ethyl lactate (1) + Na_2_Tartrate or Na_3_Citrate or K_3_Citrate (2) + water (3)} at 298.15 K and 0.1 MPa.

Tie Line	Phase	*C*/g·mL^−1^	*K*	TLL/% [24,25]
		**Epicatechin–Na_2_Tartrate**		
**1**	Top	2.75 × 10^−5^	2.12	38.33
	Bottom	1.30 × 10^−5^		
**2**	Top	2.70 × 10^−5^	2.56	49.02
	Bottom	1.06 × 10^−5^		
**3**	Top	3.00 × 10^−5^	3.71	57.82
	Bottom	8.09 × 10^−6^		
**4**	Top	3.04 × 10^−5^	4.10	66.14
	Bottom	7.43 × 10^−6^		
**5**	Top	2.87 × 10^−5^	5.43	70.90
	Bottom	5.29 × 10^−6^		
		**Epicatechin–K_3_Citrate**		
**1**	Top	3.20 × 10^−5^	4.75	45.91
	Bottom	6.73 × 10^−6^		
**2**	Top	3.21 × 10^−5^	6.97	54.02
	Bottom	4.60 × 10^−6^		
**3**	Top	3.26 × 10^−5^	12.28	62.96
	Bottom	2.65 × 10^−6^		
**4**	Top	3.23 × 10^−5^	16.09	68.28
	Bottom	2.01 × 10^−6^		
**5**	Top	3.14 × 10^−5^	21.43	73.92
	Bottom	1.47 × 10^−6^		
**6**	Top	3.04 × 10^−5^	24.86	77.66
	Bottom	1.22 × 10^−6^		
		**Vitamin B12–Na_2_Tartrate**		
**1**	Top	6.15 × 10^−4^	1.87	38.33
	Bottom	6.11× 10^−4^		
**2**	Top	7.79 × 10^−4^	2.74	49.02
	Bottom	4.38 × 10^−4^		
**3**	Top	1.01 × 10^−3^	4.91	57.82
	Bottom	2.22 × 10^−4^		
**4**	Top	1.14 × 10^−3^	10.35	66.14
	Bottom	9.53 × 10^−5^		
**5**	Top	1.18 × 10^−3^	15.96	70.90
	Bottom	5.44 × 10^−5^		
		**Vitamin B12–K_3_Citrate**		
**1**	Top	2.31 × 10^−4^	5.26	45.91
	Bottom	4.39 × 10^−5^		
**2**	Top	2.30 × 10^−4^	8.80	54.02
	Bottom	2.61 × 10^−5^		
**3**	Top	2.40 × 10^−4^	17.33	62.96
	Bottom	1.38 × 10^−5^		
**4**	Top	2.34 × 10^−4^	24.88	68.28
	Bottom	9.40 × 10^−6^		
**5**	Top	2.34 × 10^−4^	46.05	73.92
	Bottom	5.09 × 10^−6^		
**6**	Top	2.30 × 10^−4^	78.56	77.66
	Bottom	2.92 × 10^−6^		
		**Nicotinic acid–Na_2_Tartrate**		
**1**	Top	3.15 × 10^−4^	1.41	38.33
	Bottom	2.24 × 10^−4^		
**2**	Top	3.30 × 10^−4^	1.66	49.02
	Bottom	1.99 × 10^−4^		
**3**	Top	3.45 × 10^−4^	1.91	57.82
	Bottom	1.81 × 10^−4^		
**4**	Top	3.52 × 10^−4^	2.30	66.14
	Bottom	1.53 × 10^−4^		
**5**	Top	3.51 × 10^−4^	2.60	70.90
	Bottom	1.35 × 10^−4^		
		**Nicotinic acid–Na_3_Citrate**		
**1**	Top	3.23 × 10^−4^	1.67	37.85
	Bottom	1.94 × 10^−4^		
**2**	Top	3.47 × 10^−4^	2.04	48.18
	Bottom	1.70 × 10^−4^		
**3**	Top	3.46 × 10^−4^	2.28	55.17
	Bottom	1.52 × 10^−4^		
**4**	Top	3.46 × 10^−4^	2.62	61.17
	Bottom	1.32 × 10^−4^		
**5**	Top	3.48 × 10^−4^	2.86	65.44
	Bottom	1.22 × 10^−4^		
**6**	Top	3.67 × 10^−4^	3.55	69.68
	Bottom	1.03 × 10^−4^		

**Table 6:** Calculated solute losses ($L_{s}$), extraction efficiency (*E*) intervals, and tie-line lengths (TLL) for the extraction of epicatechin, cyanocobalamin, and nicotinic acid in the ATPS {ethyl lactate (1) + Na_2_Tartrate or Na_3_Citrate or K_3_Citrate (2) + water (3)} at 298.15 K and 0.1 MPa.

Tie line	$L_{s}$/%	*E*/%	TLL/% [24,25]
	**Epicatechin—Na_2_Tartrate**		
**1**	−1.12	55.7–56.9	38.33
**2**	−2.37	67.2–69.6	49.02
**3**	−4.11	73.5–77.6	57.82
**4**	−1.21	79.2–80.4	66.14
**5**	−4.41	83.4–87.8	70.90
	**Epicatechin–K_3_Citrate**		
**1**	−1.69	79.4–81.1	45.91
**2**	−2.04	86.3–88.4	54.02
**3**	−1.83	91.9–93.7	62.96
**4**	−1.55	93.8–95.3	68.28
**5**	−1.20	95.6–96.8	73.92
**6**	−0.40	97.0–97.5	77.66
	**Vitamin B12–Na_2_Tartrate**		
**1**	−1.86	49.2–51.1	38.33
**2**	−2.48	62.5–64.9	49.02
**3**	−2.08	80.2–82.3	57.82
**4**	−1.37	91.0–92.4	66.14
**5**	−0.88	94.8–95.6	70.90
	Vitamin B12–K_3_Citrate		
**1**	−2.37	81.6–84.0	45.91
**2**	−1.61	89.6–91.2	54.02
**3**	−1.33	94.1–95.5	62.96
**4**	−2.39	94.7–97.1	68.28
**5**	−1.31	97.2–98.5	73.92
**6**	−1.68	97.5–99.2	77.66
	**Nicotinic acid–Na_2_Tartrate**		
**1**	−0.91	39.9–40.8	38.33
**2**	−2.36	47.1–49.5	49.02
**3**	−2.71	56.2–59.0	57.82
**4**	−2.93	66.6–69.5	66.14
**5**	−2.92	72.7–75.7	70.90
	**Nicotinic acid–Na_3_Citrate**		
**1**	−2.32	48.7–51.0	37.85
**2**	−2.38	59.2–61.6	48.18
**3**	−2.49	65.8–68.3	55.17
**4**	−4.94	69.1–74.0	61.17
**5**	−4.76	72.8–77.6	65.44
**6**	−1.60	80.1–81.7	69.68

## Data Availability

The data presented in this study are available on request from the corresponding author.

## Supplementary Materials

The following supporting information can be downloaded at https://www.mdpi.com/article/10.3390/molecules27227838/s1: Figure S1: Influence of pH in the UV-Vis absorbance spectra of vitamin B12 at 298.15 K and 0.1 MPa; Figure S2: Influence of pH in the UV-Vis absorbance spectra of nicotinic acid at 298.15 K and 0.1 MPa; Figure S3: UV-Vis absorbance spectra of the aqueous stock solution of epicatechin in the moment of preparation and after 3 days of settling at 298.15 K and 0.1 MPa; Figure S4: UV-Vis absorbance spectra of the aqueous stock solution of vitamin B12 in the moment of preparation and after 3 days of settling at 298.15 K and 0.1 MPa; Figure S5: UV-Vis absorbance spectra of the aqueous stock solution of nicotinic acid in the moment of preparation and after 3 days of settling at 298.15 K and 0.1 MPa; Figure S6: Influence of the tie-line compositions in the fraction of the biomolecule stages of nicotinic acid in the ATPS {ethyl lactate (1) + trisodium citrate (2) + water (3)} at 298.15 K and 0.1 MPa; Figure S7: Influence of the tie-line compositions in the fraction of the biomolecule stages of epicatechin and cyanocobalamin (vitamin B12) in the ATPS {ethyl lactate (1) + tripotassium citrate (2) + water (3)} at 298.15 K and 0.1 MPa; Table S1: Calculated fractions of each biomolecule stage and mean electrical charge (*q*) at different pH values for epicatechin; Table S2: Calculated fractions of each biomolecule stage and mean electrical charge (*q*) at different pH values for vitamin B12; Table S3: Calculated fractions of each biomolecule stage and mean electrical charge (*q*) at different pH values for nicotinic acid.
