# Supplementary material for: Extraction of Polyphenols and Vitamins Using Biodegradable ATPS Based on Ethyl Lactate
Source: Molecules. 2022 Nov 14;27(22):7838. doi: 10.3390/molecules27227838 (PMC9698203; doi:10.3390/molecules27227838)
Supplement: Supplementary file 1 [file molecules-27-07838-s001.zip › molecules-1964509-supplementary.pdf]

Supplementary Materials

Extraction of polyphenols and vitamins using biodegradable ATPS based on ethyl lactate

Pedro Velho [1,2], Luís Marques [1,2], Eugénia A. Macedo [1,2,*]

[1] LSRE-LCM - Laboratory of Separation and Reaction Engineering – Laboratory of Catalysis and Materials, Faculty of Engineering, University of Porto, Rua Dr. Roberto Frias, 4200-465 Porto, Portugal.

[2] ALiCE - Associate Laboratory in Chemical Engineering, Faculty of Engineering, University of Porto, Rua Dr. Roberto Frias, 4200-465 Porto, Portugal.

*Corresponding author. Phone: +351 220 411 653. E-mail: eamacedo@fe.up.pt

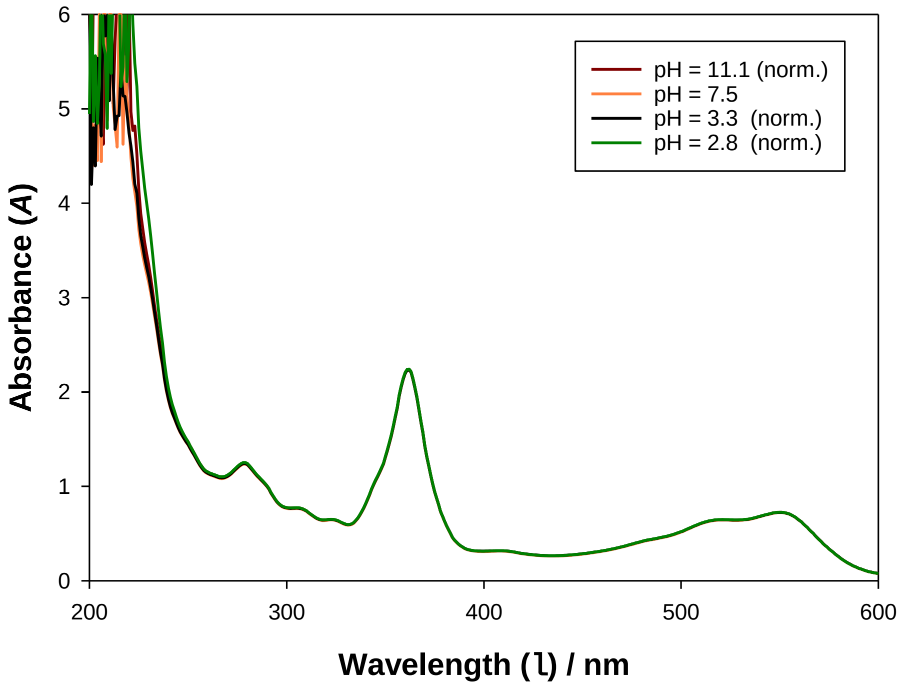

Figure S1 - Influence of pH in the UV-Vis absorbance spectra of vitamin B12 ($3.12\cdot 10^{-4}$ g·mL^{-1}) at 298.15 K and 0.1 MPa.

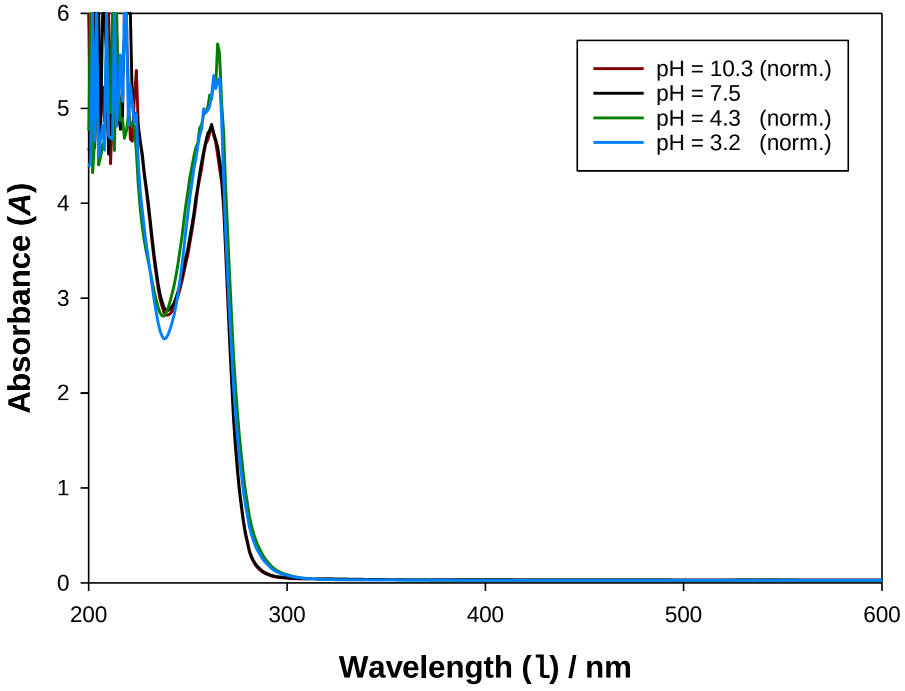

Figure S2 - Influence of pH in the UV-Vis absorbance spectra of nicotinic acid ($2.50\cdot10^{-4}$ g·mL^{-1} and pH = 7.5) at 298.15 K and 0.1 MPa.

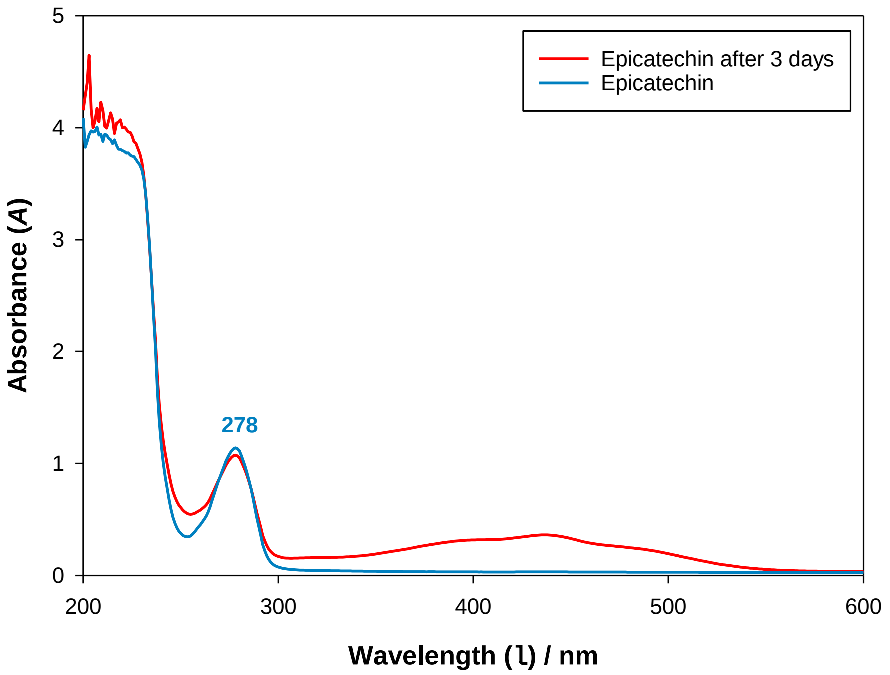

Figure S3 – UV-Vis absorbance spectra of the aqueous stock solution of epicatechin ($1.54{\cdot}10^{-4}$ g·mL^{-1} and pH = 7.5) in the moment of preparation and after 3 days of settling at 298.15 K and 0.1 MPa.

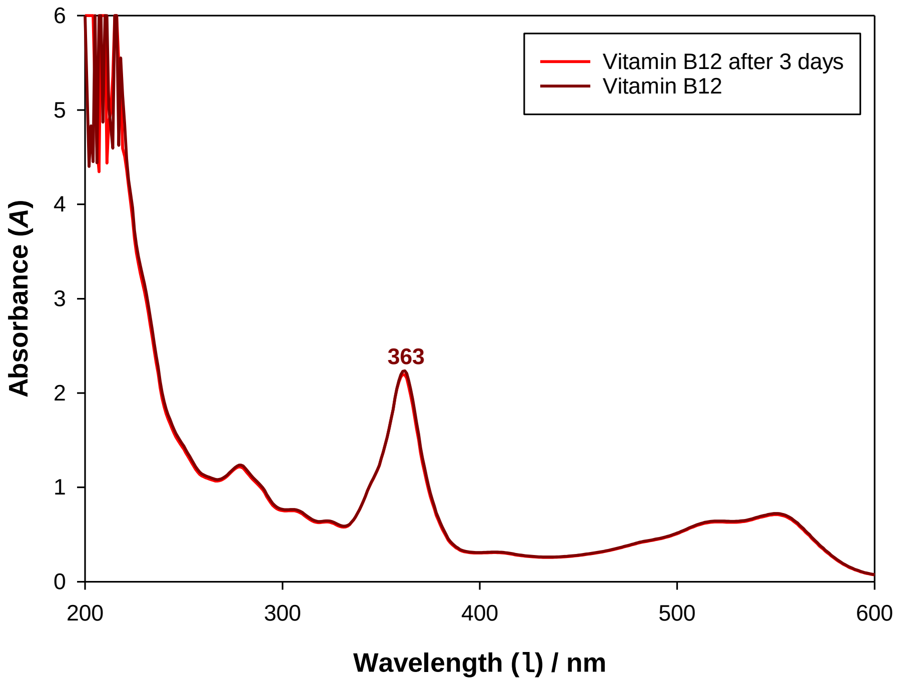

Figure S4 – UV-Vis absorbance spectra of the aqueous stock solution of vitamin B12 ($3.12{\cdot}10^{-4}$ g·mL^{-1} and pH = 7.5) in the moment of preparation and after 3 days of settling at 298.15 K and 0.1 MPa.

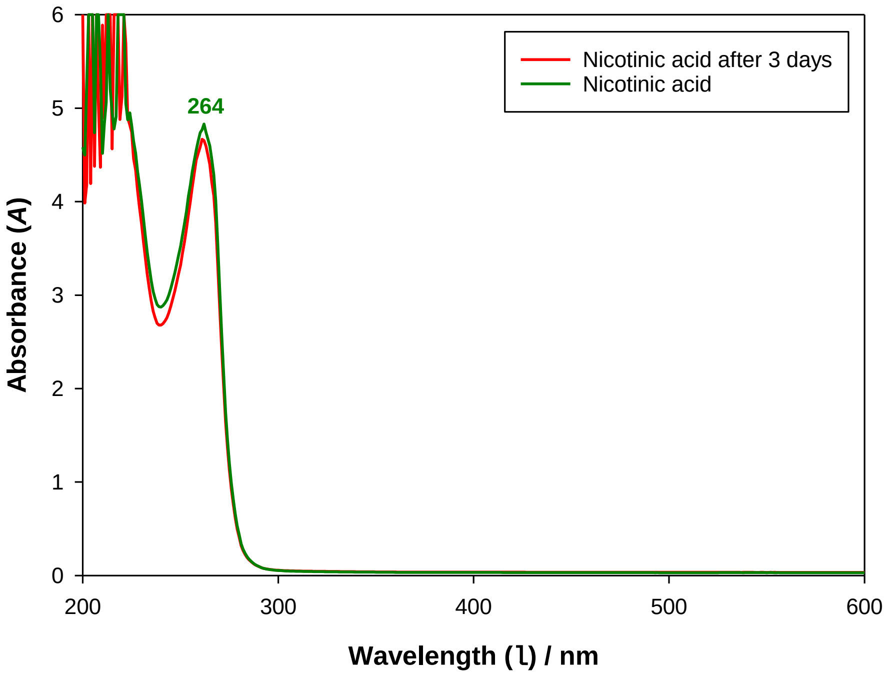

Figure S5 – UV-Vis absorbance spectra of the aqueous stock solution of nicotinic acid ($2.50 \cdot 10^{-4}$ $g \cdot mL^{-1}$ and pH = 7.5) in the moment of preparation and after 3 days of settling at 298.15 K and 0.1 MPa.

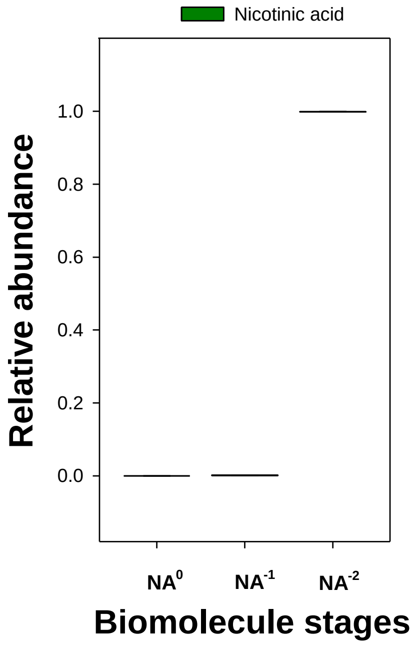

Figure S6 - Influence of the tie-line compositions in the fraction of the biomolecule stages of nicotinic acid in the ATPS {ethyl lactate (1) + trisodium citrate (2) + water (3)} at 298.15 K and 0.1 MPa. NA^{0}, NA^{-1} and NA^{-2} stand for the biomolecule stages of nicotinic acid with electrical charges equal to 0, -1 and -2 e, respectively, and e stands for the elementary charge ($1.602 \cdot 10^{-19}$ C).

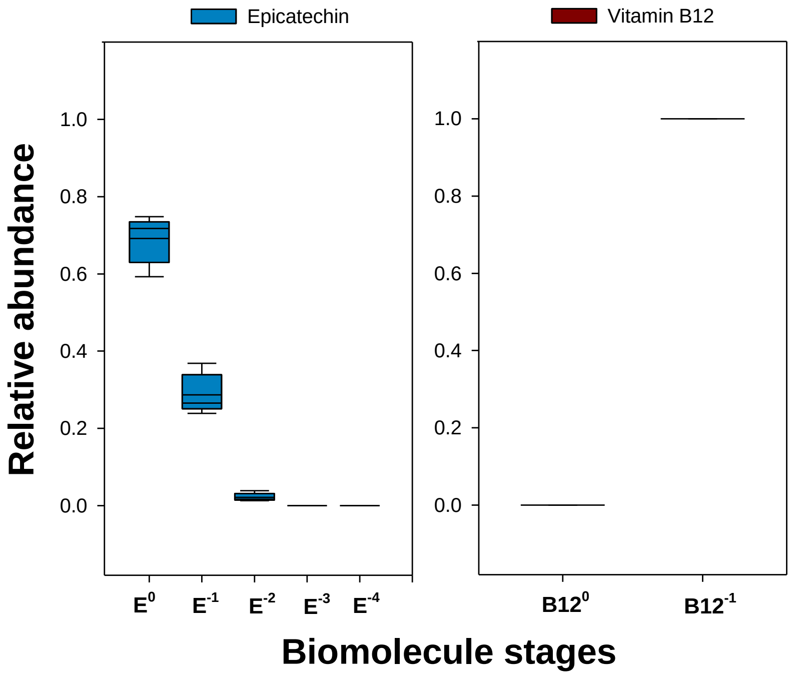

Figure S7 - Influence of the tie-line compositions in the fraction of the biomolecule stages of epicatechin and cyanocobalamin (vitamin B12) in the ATPS {ethyl lactate (1) + tripotassium citrate (2) + water (3)} at 298.15 K and 0.1 MPa. E^0, E^{-1}, E^{-2}, E^{-3} and E^{-4} stand for the biomolecule stages of epicatechin with electrical charges equal to 0, -1, -2, -3 and -4 e, respectively; $B12^0$ and $B12^{-1}$ stand for the biomolecule stages of cyanocobalamin with electrical charges equal to 0 and -1 e, respectively, and e stands for the elementary charge ($1.602 \cdot 10^{-19}$ C).

Table S1: Calculated fractions of each biomolecule stage and mean electrical charge (q) at different pH values for epicatechin.

pH	x_{E^0} [a]	$x_{E^{-1}}$	$x_{E^{-2}}$	$x_{E^{-3}}$	$x_{E^{-4}}$	q / e
0.00	1.00	0.00	0.00	0.00	0.00	0.00
0.25	1.00	0.00	0.00	0.00	0.00	0.00
0.50	1.00	0.00	0.00	0.00	0.00	0.00
0.75	1.00	0.00	0.00	0.00	0.00	0.00
1.00	1.00	0.00	0.00	0.00	0.00	0.00
1.25	1.00	0.00	0.00	0.00	0.00	0.00
1.50	1.00	0.00	0.00	0.00	0.00	0.00
1.75	1.00	0.00	0.00	0.00	0.00	0.00
2.00	1.00	0.00	0.00	0.00	0.00	0.00
2.25	1.00	0.00	0.00	0.00	0.00	0.00
2.50	1.00	0.00	0.00	0.00	0.00	0.00
2.75	1.00	0.00	0.00	0.00	0.00	0.00
3.00	1.00	0.00	0.00	0.00	0.00	0.00
3.25	1.00	0.00	0.00	0.00	0.00	0.00
3.50	1.00	0.00	0.00	0.00	0.00	0.00
3.75	1.00	0.00	0.00	0.00	0.00	0.00
4.00	1.00	0.00	0.00	0.00	0.00	0.00
4.25	1.00	0.00	0.00	0.00	0.00	0.00
4.50	1.00	0.00	0.00	0.00	0.00	0.00
4.75	1.00	0.00	0.00	0.00	0.00	0.00
5.00	1.00	0.00	0.00	0.00	0.00	0.00
5.25	1.00	0.00	0.00	0.00	0.00	0.00
5.50	1.00	0.00	0.00	0.00	0.00	0.00
5.75	1.00	0.00	0.00	0.00	0.00	0.00
6.00	1.00	0.00	0.00	0.00	0.00	0.00
6.25	1.00	0.00	0.00	0.00	0.00	0.00
6.50	0.99	0.01	0.00	0.00	0.00	-0.01
6.75	0.99	0.01	0.00	0.00	0.00	-0.01
7.00	0.98	0.02	0.00	0.00	0.00	-0.02
7.25	0.97	0.03	0.00	0.00	0.00	-0.03
7.50	0.94	0.06	0.00	0.00	0.00	-0.06
7.75	0.90	0.10	0.00	0.00	0.00	-0.10
8.00	0.84	0.16	0.01	0.00	0.00	-0.17
8.25	0.74	0.25	0.01	0.00	0.00	-0.28
8.50	0.60	0.36	0.04	0.00	0.00	-0.44
8.75	0.44	0.47	0.09	0.00	0.00	-0.65
9.00	0.28	0.54	0.17	0.00	0.00	-0.89
9.25	0.16	0.53	0.31	0.00	0.00	-1.16
9.50	0.08	0.45	0.46	0.01	0.00	-1.41
9.75	0.03	0.34	0.61	0.02	0.00	-1.62
10.00	0.01	0.22	0.72	0.04	0.00	-1.80
10.25	0.00	0.14	0.78	0.08	0.00	-1.94
10.50	0.00	0.08	0.78	0.14	0.00	-2.07
10.75	0.00	0.04	0.72	0.24	0.00	-2.20
11.00	0.00	0.02	0.62	0.36	0.00	-2.35
11.25	0.00	0.01	0.48	0.51	0.00	-2.50
11.50	0.00	0.00	0.35	0.64	0.01	-2.66
11.75	0.00	0.00	0.23	0.75	0.02	-2.79
12.00	0.00	0.00	0.14	0.83	0.03	-2.89
12.25	0.00	0.00	0.08	0.86	0.06	-2.98
12.50	0.00	0.00	0.05	0.85	0.11	-3.06
12.75	0.00	0.00	0.02	0.80	0.18	-3.15
13.00	0.00	0.00	0.01	0.71	0.28	-3.27
13.25	0.00	0.00	0.01	0.58	0.41	-3.41
13.50	0.00	0.00	0.00	0.44	0.56	-3.55
13.75	0.00	0.00	0.00	0.31	0.69	-3.69
14.00	0.00	0.00	0.00	0.20	0.80	-3.80

[a] x_{E^0}, $x_{E^{-1}}$, $x_{E^{-2}}$, $x_{E^{-3}}$ and $x_{E^{-4}}$ are the fractions of epicatechin in the biomolecule stages of electrical charges equal to 0, -1, -2, -3 and -4 e, respectively. e stands for the elementary charge ($1.602 \cdot 10^{-19}$ C).

Table S2: Calculated fractions of each biomolecule stage and mean electrical charge (q) at different pH values for vitamin B12.

pH	x_{B12^0} [a]	$x_{B12^{-1}}$	q / e
0.00	1.00	0.00	0.00
0.25	1.00	0.00	0.00
0.50	1.00	0.00	0.00
0.75	1.00	0.00	0.00
1.00	0.99	0.01	-0.01
1.25	0.99	0.01	-0.01
1.50	0.98	0.02	-0.02
1.75	0.97	0.03	-0.03
2.00	0.95	0.05	-0.05
2.25	0.91	0.09	-0.09
2.50	0.86	0.14	-0.14
2.75	0.77	0.23	-0.23
3.00	0.66	0.34	-0.34
3.25	0.52	0.48	-0.48
3.50	0.38	0.62	-0.62
3.75	0.25	0.75	-0.75
4.00	0.16	0.84	-0.84
4.25	0.10	0.90	-0.90
4.50	0.06	0.94	-0.94
4.75	0.03	0.97	-0.97
5.00	0.02	0.98	-0.98
5.25	0.01	0.99	-0.99
5.50	0.01	0.99	-0.99
5.75	0.00	1.00	-1.00
6.00	0.00	1.00	-1.00
6.25	0.00	1.00	-1.00
6.50	0.00	1.00	-1.00
6.75	0.00	1.00	-1.00
7.00	0.00	1.00	-1.00
7.25	0.00	1.00	-1.00
7.50	0.00	1.00	-1.00
7.75	0.00	1.00	-1.00
8.00	0.00	1.00	-1.00
8.25	0.00	1.00	-1.00
8.50	0.00	1.00	-1.00
8.75	0.00	1.00	-1.00
9.00	0.00	1.00	-1.00
9.25	0.00	1.00	-1.00
9.50	0.00	1.00	-1.00
9.75	0.00	1.00	-1.00
10.00	0.00	1.00	-1.00
10.25	0.00	1.00	-1.00
10.50	0.00	1.00	-1.00
10.75	0.00	1.00	-1.00
11.00	0.00	1.00	-1.00
11.25	0.00	1.00	-1.00
11.50	0.00	1.00	-1.00
11.75	0.00	1.00	-1.00
12.00	0.00	1.00	-1.00
12.25	0.00	1.00	-1.00
12.50	0.00	1.00	-1.00
12.75	0.00	1.00	-1.00
13.00	0.00	1.00	-1.00
13.25	0.00	1.00	-1.00
13.50	0.00	1.00	-1.00
13.75	0.00	1.00	-1.00
14.00	0.00	1.00	-1.00

[a] x_{B12^0}, and $x_{B12^{-1}}$ are the fractions of vitamin B12 in the biomolecule stages of electrical charges equal to 0 and -1 e, respectively. e stands for the elementary charge ($1.602 \cdot 10^{-19}$ C).

Table S3: Calculated fractions of each biomolecule stage and mean electrical charge (q) at different pH values for nicotinic acid.

**pH**	x_{NA^0} [a]	$x_{\mathrm{NA}^{-1}}$	$x_{\mathrm{NA}^{-2}}$	q / **e**
0.00	0.99	0.01	0.00	-0.01
0.25	0.98	0.02	0.00	-0.02
0.50	0.97	0.03	0.00	-0.03
0.75	0.95	0.05	0.00	-0.05
1.00	0.91	0.09	0.00	-0.09
1.25	0.85	0.15	0.00	-0.15
1.50	0.76	0.24	0.00	-0.24
1.75	0.64	0.36	0.00	-0.36
2.00	0.50	0.50	0.00	-0.50
2.25	0.36	0.64	0.00	-0.64
2.50	0.24	0.76	0.00	-0.76
2.75	0.15	0.84	0.01	-0.86
3.00	0.09	0.90	0.01	-0.92
3.25	0.05	0.92	0.02	-0.97
3.50	0.03	0.93	0.04	-1.02
3.75	0.02	0.91	0.08	-1.06
4.00	0.01	0.86	0.13	-1.12
4.25	0.00	0.78	0.21	-1.21
4.50	0.00	0.67	0.32	-1.32
4.75	0.00	0.54	0.46	-1.46
5.00	0.00	0.40	0.60	-1.60
5.25	0.00	0.27	0.73	-1.73
5.50	0.00	0.17	0.83	-1.83
5.75	0.00	0.11	0.89	-1.89
6.00	0.00	0.06	0.94	-1.94
6.25	0.00	0.04	0.96	-1.96
6.50	0.00	0.02	0.98	-1.98
6.75	0.00	0.01	0.99	-1.99
7.00	0.00	0.01	0.99	-1.99
7.25	0.00	0.00	1.00	-2.00
7.50	0.00	0.00	1.00	-2.00
7.75	0.00	0.00	1.00	-2.00
8.00	0.00	0.00	1.00	-2.00
8.25	0.00	0.00	1.00	-2.00
8.50	0.00	0.00	1.00	-2.00
8.75	0.00	0.00	1.00	-2.00
9.00	0.00	0.00	1.00	-2.00
9.25	0.00	0.00	1.00	-2.00
9.50	0.00	0.00	1.00	-2.00
9.75	0.00	0.00	1.00	-2.00
10.00	0.00	0.00	1.00	-2.00
10.25	0.00	0.00	1.00	-2.00
10.50	0.00	0.00	1.00	-2.00
10.75	0.00	0.00	1.00	-2.00
11.00	0.00	0.00	1.00	-2.00
11.25	0.00	0.00	1.00	-2.00
11.50	0.00	0.00	1.00	-2.00
11.75	0.00	0.00	1.00	-2.00
12.00	0.00	0.00	1.00	-2.00
12.25	0.00	0.00	1.00	-2.00
12.50	0.00	0.00	1.00	-2.00
12.75	0.00	0.00	1.00	-2.00
13.00	0.00	0.00	1.00	-2.00
13.25	0.00	0.00	1.00	-2.00
13.50	0.00	0.00	1.00	-2.00
13.75	0.00	0.00	1.00	-2.00
14.00	0.00	0.00	1.00	-2.00

[a] x_{NA^0}, $x_{\mathrm{NA}^{-1}}$, and $x_{\mathrm{NA}^{-2}}$ are the fractions of nicotinic acid in the biomolecule stages of electrical charges equal to 0, -1 and -2 e, respectively. e stands for the elementary charge ($1.602 \cdot 10^{-19}$ C).
